# Major depressive disorders related to Post-traumatic cases after cervical spine injuries in amateur athletes

**DOI:** 10.1192/j.eurpsy.2026.11394

**Published:** 2026-07-31

**Authors:** N. Syrmos

**Affiliations:** Aristotle University, Thessaloniki, Greece

## Abstract

**Introduction:**

Introduction- Major depressive disorders in combination Post-traumatic stress disorder remains mental pathological situations in which the cause is readily identifiable. The two disorders (MDD and PTSD) represent distinct constructs and depend, at least in part, on separate biological mechanisms. They are a related to stress psychopathology.

**Objectives:**

Aim-Aim of this study is to present cases with major deppresive disorders and post-traumatic stress disorders cases in amateur athletes after cervical spine injuries.

**Methods:**

Material-Methods-20 patients are presented. 16 males (80%) and 4 females (20%) Range of age between 18-48 years old . All of theme (20-100%) were evaluated, with clinical and radiological exam(ct,mri) plus with the Diagnostic and Statistical Manual of Mental Disorders (DSM-5) diagnostic criteria, prevalence, and presentation of patients with PTSD.

**Results:**

Results-Conlusions-Good results in 15 cases (75%) and moderate in 5 (25%). Collaboration between various medical disciplines is essential in order to achive optimal results and clarify the theory,the etiology, and treatment effectiveness..

**Conclusions:**

Conlusions-Recommendations regarding effective psychotherapy, neurological,psychiatric and pharmacotherapy, emerging treatment, and accurate management are necessary in order to achieve optimal overall treatment.

**Disclosure of Interest:**

None Declared

